# Current Concepts on the Physiopathological Relevance of Dopaminergic Receptors

**DOI:** 10.3389/fncel.2017.00027

**Published:** 2017-02-08

**Authors:** Ada Ledonne, Nicola B. Mercuri

**Affiliations:** ^1^Department of Experimental Neuroscience, Santa Lucia FoundationRome, Italy; ^2^Department of Systems Medicine, University of Rome “Tor Vergata”Rome, Italy

**Keywords:** dopamine, DAergic receptors, nigrostriatal pathway, mesolimbic pathway, mesocortical pathway

## Abstract

Dopamine (DA) is a key neurotransmitter modulating essential functions of the central nervous system (CNS), like voluntary movement, reward, several cognitive functions and goal-oriented behaviors. The factual relevance of DAergic transmission can be well appreciated by considering that its dysfunction is recognized as a core alteration in several devastating neurological and psychiatric disorders, including Parkinson’s disease (PD) and associated movement disorders, as well as, schizophrenia, bipolar disorder, attention deficit hyperactivity disorder (ADHD) and addiction. Here we present an overview of the current knowledge on the involvement of DAergic receptors in the regulation of key physiological brain activities, and the consequences of their dysfunctions in brain disorders such as PD, schizophrenia and addiction.

## Introduction

Dopamine (DA) regulates important physiological brain’s functions, including locomotion, reward and cognition, through different DAergic pathways, mainly originating in two mesencephalic nuclei, the ventral tegmental area (VTA) and the substantia nigra pars compacta (SNpc; Dahlström and Fuxe, 1964). DAergic neurons of the VTA project to limbic areas (nucleus accumbens (NAc), hippocampus and amygdala) and cortical regions, thus composing the mesolimbic- and mesocortical pathways, respectively, those of the SNpc constitute the nigrostriatal pathway mainly projecting to the dorsal striatum.

According to a traditional belief, the different DAergic pathways mediate specific physiological functions, with the nigrostriatal pathway involved in locomotion, and the mesolimbic/mesocortical pathways implicated in reward and cognition. The evidence that Parkinson’s disease (PD), a disorder mainly characterized by motor inabilities, is primarily due to a dysfunction of nigrostriatal pathway, whereas neuropsychiatric disorders, like schizophrenia and addiction, involve a major dysregulation of mesolimbic/mesocortical pathways, reinforces the hypothesis of a functional segregation of DAergic pathways. Nowadays, however, this functional/physiopathological subdivision is outdated, since a key role of the nigrostriatal pathway has been recognized in cognitive functions (Haber, 2014), in reward, craving and aversion (Wise, 2009), and in schizophrenia (Perez-Costas et al., 2010; Yoon et al., 2013; Weinstein et al., 2017).

## DAergic Receptors

### Classification, Signaling and Regulatory Mechanisms

**Figure 1:**
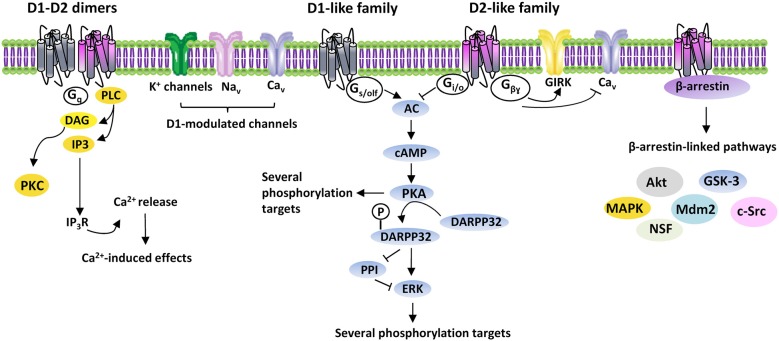
**DAergic receptors signaling.** Intracellular signaling pathways activated by D1- and D2-like receptors families. G_s/olf_, G_i/o_, G_q_, G_βγ_, G proteins; AC, adenylyl cyclase; cAMP, 3′-5′-cyclic adenosine monophosphate; DARPP32, cyclic AMP-regulated phosphoprotein, 32 kDa; PPI, protein-phosphatase 1; ERK, extracellular signal-regulated kinase protein kinase; PLC, phospholipase C; DAG, diacylglycerol; PKC, protein kinase C; IP_3_, inositol triphosphate; IP_3_R, inositol triphosphate receptor; Ca^2+^, calcium; GIRK, G-protein gated inwardly rectifying K^+^ channels; Ca_v_, voltage-activated Ca^2+^ channels; Na_v_, voltage-activated Na^+^ channels; Akt, thymoma viral proto-oncogene; GSK-3, Glycogen Synthase Kinase-3; MAPK, mitogen-activated protein kinase; Mdm2, mouse double minute 2 homolog; c-Src, proto-oncogene non-receptor tyrosine kinase; NSF, N-ethylmaleimide-sensitive factor.

DA-induced effects are mediated by five G protein-coupled receptors (GPCR), classified into two subclasses: the D1R-like and D2R-like receptor families. D1R-like receptors (D1R and D5R) are coupled to G_s/olf_ proteins and stimulate adenylate cyclase (AC), with production of cyclic adenosine monophosphate (cAMP) and activation of cAMP-dependent pathways, mainly including protein kinase A (PKA) and other downstream signals. D1R modulate different ionic channels, including voltage-activated Na^+^- (Na_v_), K^+^- (K_v_) and Ca^2+^ (Ca_v_) channels, Ca^2+^-activated K^+^- (K_Ca_) and G-protein gated inwardly rectifying K^+^ (GIRK) channels (Maurice et al., 2001; Witkowski et al., 2008; Yang et al., 2013). D2R-like receptors (D2R, D3R and D4R), by coupling to G_i/o_ proteins, induce inhibition of AC and PKA-dependent pathways, as well as activation of GIRK and closure of Ca_V_ (Missale et al., 1998) (Figure 1).

D2R-like receptors genes generate variants. D2R exists in two functional isoforms, D2 long (D2L) and D2 short (D2S; Giros et al., 1989), whereas several D3R isoforms have been identified (Giros et al., 1991). Multiple D4R variants are produced, mostly having a domain repeated 2 (2R), 4 (4R) and 7 (7R) times (Van Tol et al., 1992).

In addition to act as monomers, DAergic receptors constitute dimeric and/or oligomeric complexes by association of different subtypes either alone or with other GPCRs and ligand-gated channels. Homodimers exist, like D1R-D2R, D2R-D4R, D1R-D3R, D2R-D3R and D2R-D5R, as well as oligomeric complexes containing DAergic receptors associated to the adenosine A1 and A2, serotoninergic 5-HT_2A_, histaminergic H3, glutamatergic mGlu5 and NMDA receptors (Perreault et al., 2014). Dimeric/oligomeric complexes increase the complexity of DA-mediated effects, since they may display pharmacological/functional properties distinct from their constituent receptors. Indeed, D1R-D2R are linked to G_q/11_ proteins, thus modulating phospholipase C (PLC), which produces inositol trisphosphate (IP_3_) and diacylglycerol (DAG) to regulate intracellular Ca^2+^ (Lee et al., 2004) (Figure 1).

While the prevailing belief is that DAergic receptors act through G proteins, they can also activate G proteins-independent mechanisms. A role in the G protein-independent signaling is played by arrestins, multifunctional adaptor proteins, which bind DAergic receptors phosphorylated by GPCR kinases (GRKs; Gainetdinov et al., 2004). Binding of arrestins recruits several proteins, including Akt, GSK-3, MAPK, c-Src, Mdm2 and N-ethylmaleimide-sensitive factor, thus greatly enhancing DA-activated pathways (Beaulieu and Gainetdinov, 2011) (Figure 1).

GRKs also regulate DAergic receptors by mediating their desensitization, since their phosphorylation elicits receptor’s endocytosis. GRK2, GRK3, GRK4, GRK5 and GRK6 regulate D1R and D2R (Tiberi et al., 1996; Ito et al., 1999; Watanabe et al., 2002; Villar et al., 2009), whereas GRK4 controls D3R (Villar et al., 2009).

Besides GRKs, the regulators of G protein signaling (RGS), a group of GTPase-activating proteins acting on G protein, negatively modulate DAergic receptors. Among RGS members, RGS9–2 affects D2R (Cabrera-Vera et al., 2004), RGS4 modulates D2R, D1R/D3R (Min et al., 2012) and D2R/A2A (Lerner and Kreitzer, 2012), whereas R7 subgroup regulate D2R (Wani et al., 2012).

## Physiological Functions of DAergic Receptors

### Control of Locomotion

The regulation of locomotion represents a well-characterized function of DAergic receptors. Thus, DA in the dorsal striatum modulates basal ganglia activity, by DAergic receptors mainly expressed on GABAergic medium spiny neurons (MSNs). D1R and D2R principally display a segregated expression on striatal MSNs (Gerfen and Surmeier, 2011). D1R-expressing MSNs directly project to basal ganglia’s output nuclei, the substantia nigra pars reticulata (SNpr) and the globus pallidus internus (GPi; direct pathway). D2R-expressing MSNs project to intermediate nuclei, the external globus pallidus (GPe) that projects to the subthalamic nucleus (STN; indirect pathway). The combined D1R/D2R activation regulates SNpr/GPi, which control the excitation of the cortex via thalamus, thus promoting/inhibiting locomotion. An opposite role of D1R- and D2R-expressing neurons on movement has been recently confirmed, since the stimulation of direct pathway facilitates movement, while the activation of indirect pathway causes hypokinesia (Kravitz et al., 2010). However, the evidence that D1R/D2R is co-expressed in a small subgroup of MSNs (Aizman et al., 2000) adds complexity to their roles in movement.

Striatal DA effects are regulated by D2R autoreceptors, localized presynaptically on DAergic terminals and mesencephalic cells. Thus, D2R provide a negative feedback, which by tuning neuronal firing and DA synthesis/release, changes extracellular neurotransmitter level (Lacey et al., 1987; Wolf and Roth, 1990). D3R, possibly as D2R/D3R, could also act as inhibitory autoreceptors (De Mei et al., 2009; but see Mercuri et al., 1997). Accordingly to an important role of D2R in movement regulation, the constitutive deletion of all D2R (Kelly et al., 1998) or D2L isoforms (Usiello et al., 2000; Wang et al., 2000), as well as D2R deletion in adulthood (Bello et al., 2016) impairs spontaneous and agonists-stimulated locomotion. Interestingly, D2R on striatal cholinergic interneurons are involved in catalexia induced by neuroleptics (D2 antagonists; Kharkwal et al., 2016).

Specific DA-activated pathways, such as MEK/ERK, GSK3β and mTOR, have been implicated in locomotion (Beaulieu et al., 2005, 2007; Santini et al., 2007; Urs et al., 2011). Moreover, GRK2 might regulate locomotion, since GRK2 KO mice display altered movements associated with DAergic dysfunctions (Daigle et al., 2014).

Therefore, a proper locomotion depends on MSNs D1R and D2R function. While D2R and D3R autoreceptors mainly regulate DA extracellular levels, D4R and D5R subtypes are dispensable for DA-induced regulation of locomotion (Missale et al., 1998).

### Reward

The mesolimbic DAergic pathway plays a central role in the processing of reward-related stimuli, which mainly increase extracellular DA levels in the NAc (Di Chiara, 2002; Volkow and Morales, 2015). Additionally, the nigrostriatal pathway also mediates reward processing (Wise, 2009). DAergic transmission contributes to the reward prediction signal, since DAergic neuronal firing enhances following unexpected/novel rewards and is inhibited if an expected reward does not materialize (Schultz et al., 1997; Schultz, 2002).

D1R or D2R either are involved in reward induced by natural stimuli (food, sex) or addictive drugs. In the NAc, D1R and D2R are largely compartmentalized on MSNs of the direct/indirect pathways, respectively (Gerfen et al., 1990). It is believed that direct/indirect pathways have different roles in reward, with the direct pathway mediating reward while the indirect one encoding aversion (Hikida et al., 2010; Kravitz et al., 2012). Thus, D1R-dependent stimulation of the direct pathway causes reward, whereas D2R-induced inhibition of indirect pathway opposes aversion and facilitates reward (Hikida et al., 2013). Actually, accumbal D1R activation is sufficient to produce reward (Caine et al., 2002), while a combined D1R/D2R activation causes maximal reward (Steinberg et al., 2014). However, the co-expression of D1R/D2R on a subpopulation of MSNs (Perreault et al., 2012) renders the functions of D1R/D2R on direct/indirect pathways more complex than is usually thought.

D3R seem less involved in reward processing, but rather affect addictive behaviors (Sokoloff and Le Foll, 2017). Contrasting evidence exist about a role for D4R in reward (Di Ciano et al., 2014), although genetic association studies found correlations between D4R variants and addictive behaviors (Ptáček et al., 2011). D5R are not involved in reward.

### Regulation of Cognitive Functions

DA regulates essential cognitive functions through DAergic receptors expressed in the prefrontal cortex (PFC), striatum and hippocampus. However, the role of DA in cognition appears rather complex and poorly elucidated at cellular level, with DAergic receptors controlling ionic conductances and/or synaptic plasticity on different neuronal populations (Floresco and Magyar, 2006; Arnsten et al., 2015).

D1R-like receptors modulate several aspects of cognition. D1R are highly expressed in PFC and striatum and moderately in hippocampus, whereas D5R display a similar distribution with lower levels. Thus, D1R family control working memory, behavioral flexibility, decision-making and goal-directed behaviors (Sawaguchi and Goldman-Rakic, 1991; Ragozzino, 2002; Floresco et al., 2006), as well as hippocampal-dependent learning and memory (Packard and White, 1991; Bach et al., 1999; El-Ghundi et al., 1999; Hansen and Manahan-Vaughan, 2014). D1R/D5R regulate cortical pyramidal neurons excitability and recurrent excitations within neuronal networks (Seamans and Yang, 2004) underlying executive functions, besides controlling synaptic plasticity in striatum and hippocampus (Calabresi et al., 1992b; Kerr and Wickens, 2001; Hansen and Manahan-Vaughan, 2014).

D2R are highly expressed in striatum and hippocampus and moderately in layer 5 of PFC and regulate behavioral flexibility, goal-directed behaviors and decision-making, also affecting working- and long-term memory (Ragozzino, 2002; Floresco et al., 2006; Stelzel et al., 2013; Puig and Miller, 2015). D2R-activated mechanisms modify cortical pyramidal neurons excitability (Gulledge and Jaffe, 1998; Wang and Goldman-Rakic, 2004), and/or glutamatergic synaptic plasticity in striatum and hippocampus (Calabresi et al., 1992b; Rocchetti et al., 2015; Broussard et al., 2016).

Despite D3R are almost absent in PFC, they indirectly modulate PFC-dependent cognitive functions, by inhibiting mesocortical DAergic activity and/or adjusting cortical Ach levels (Loiseau and Millan, 2009; Gross and Drescher, 2012). Thus, D3R inhibition improves attention, learning, memory and executive functions (Nakajima et al., 2013), whereas striatal D3R modulate behavioral flexibility (Groman et al., 2016).

D4R in PFC and hippocampus affect different cognitive tasks, including inhibitory avoidance and object recognition memory (Bernaerts and Tirelli, 2003; Powell et al., 2003; Woolley et al., 2008), being also involved in attention and exploratory behavior (Oak et al., 2000).

## DAergic Transmission in Brain Disorders

### Parkinson’s Disease (PD)

The progressive neurodegeneration of SNpc DAergic neurons represents the core feature of PD, a neurological disorder mainly characterized by severe motor inabilities. Indeed, striatal DAergic denervation unbalances the activation rate of direct/indirect pathways of basal ganglia, thus causing deficits in movement initiations, rigidity and bradykinesia. In PD there is a reorganization of DAergic receptors in basal ganglia (Albin et al., 1989), being D2R expression increased in MSNs-indirect pathway and D1R mRNAs reduced in MSNs-direct pathway (Gerfen et al., 1990). Moreover, the SNpc neurons degeneration leads to progressive loss of D2R on striatal presynaptic terminals. Meanwhile, as a compensation, DAergic receptors become supersensitive, possibly depending on a more effective G protein-receptors coupling and/or an increased expression of signaling proteins (Hornykiewicz, 2001; Napolitano et al., 2002). Supersensitivity has been reported for striatal D1R and D2R, particularly, in striato-pallidal/striato-nigral terminals (Corvol et al., 2004; Guigoni et al., 2007; Prieto et al., 2011). Accordingly, in PD animal models, the D1R-dependent regulation of direct pathway enhances nigral GABA release (Mango et al., 2014a).

Noteworthy, DAergic receptors supersensitivity could represent the biological substrate underlying motor abnormalities produced by prolonged treatments with the DA precursor, L-DOPA. Its administration often causes motor fluctuations and involuntary movements, namely L-DOPA-induced dyskinesia (LID). Striatal D1R hyperactivation plays a pivotal role in LID development (Cenci, 2007). Indeed, in LID animal models, D1R-linked signaling is hyperactive, with increased cAMP levels and higher phosphorylation of ERK1/2, DARPP-32 and mTOR (Greengard et al., 1999; Picconi et al., 2003; Aubert et al., 2005; Pavón et al., 2006; Santini et al., 2007). Accordingly, strategies reducing D1R functions significantly rescue motor abnormalities in LID models (Fiorentini et al., 2016). Moreover, based on D1R-A1R expression on striatal MSNs, the A1R modulation, by counteracting excessive D1R signaling in PD, reduces L-DOPA-induced involuntary movements (Mango et al., 2014b).

A dysfunctional RGS-dependent modulation of DAergic receptors could contribute to motor disorders. Indeed, RGS9–2 plays a role in the occurrence of motor anomalies in LID, since RGS9–2 KO mice develop dyskinesia associated with D2R dysfunctions, and RGS9–2 overexpression diminishes L-DOPA-induced involuntary movements (Kovoor et al., 2005; Gold et al., 2007).

### Addiction

Addiction is a neuropsychiatric disorder characterized by compulsive engagement in rewarding stimuli, despite adverse consequences. It is considered dependent on complex neuronal modifications induced by transcriptional/epigenetic mechanisms following repeated exposure to reinforcing stimuli, based on a psychobiological vulnerability (Volkow and Morales, 2015).

There are established associations between polymorphisms in DAergic receptors genes and addiction, with genetic variants of D1R, D2R, D3R and D4R linked to substance abuse, alcoholism, bulimia nervosa and pathological gambling (Blum et al., 1995; Comings et al., 1999; da Silva Lobo et al., 2007; Le Foll et al., 2009; Ptáček et al., 2011).

Modifications in the mesolimbic/mesocortical DAergic pathways represent core biological changes underlying addictive behaviors. Synthetic/natural rewards increase extracellular DA in limbic/cortical areas, besides producing other long-term modifications, including a potentiation of glutamatergic transmission in midbrain DAergic nuclei, NAc, striatum and cortex (Volkow and Morales, 2015). Moreover, long-term changes in DAergic receptor responsiveness possibly contribute to synaptic/neuronal adaptations leading to psychostimulant-induced sensitization and compulsion (Hyman et al., 2006). Actually, addictive drugs downregulate D2R-like receptors, with a reduced expression of striatal D2R and D3R in individuals addicted to cocaine, methamphetamine, alcohol or heroin (Volkow et al., 1993, 1996, 2001).

Although modifications in D1R expression have not been consistently demonstrated in addiction (Martinez et al., 2009), D1R play a prominent role in the acquisition/maintenance of self-administration behavior (Self, 2010). Thus, pharmacological/genetic D1R inhibition reduces the sensitivity to rewarding effects of psychostimulants and impairs cocaine self-administration (Caine et al., 1995, 2007).

D2R in midbrain DA neurons are also involved in the establishment of addictive behaviors, since D2R deletion enhances food intake and sensitivity to locomotor/rewarding properties of cocaine (Bello et al., 2011). Accordingly, drug intake and impulsivity are inversely correlated with D2R availability in SNpc/VTA (Buckholtz et al., 2010).

Modifications in RGS proteins affecting D2R may play a role in addiction. Psychostimulants and D2R ligands exposure alter RGS9-2 protein levels (Seeman et al., 2007), whereas amphetamine self-administration increases RGS2/RGS4 mRNAs expression in VTA/NAc, but reduces D2RS mRNAs levels in VTA (Sun et al., 2015). Moreover, RGS9-2 deletion exacerbates the rewarding/motor effects of psychostimulants, which are instead counteracted by RGS9-2 overexpression (Rahman et al., 2003; Traynor et al., 2009).

D3R do not directly control the reinforcing/psychomotor effects of psychostimulants (Reavill et al., 2000; Caine et al., 2002), although a D3R deletion increases the sensitivity to cocaine and amphetamine (Xu, 1998). D3R affect cue-induced drug-seeking behaviors and relapse (Sokoloff and Le Foll, 2017), thus D3R antagonists might represent potential therapeutics for drug addiction.

D4R is considered a minor player in mediating psychostimulants-induced reinforce (Costanza and Terry, 1998; Caine et al., 2002). D4R null mice appear more sensitive to locomotor, but not rewarding, properties of addictive drugs (Rubinstein et al., 1997; Thanos et al., 2010). Notwithstanding, *D4R* represents a susceptibility gene for food/drug dependence and pathological gambling (Comings et al., 1999; Ptáček et al., 2011; Silveira et al., 2014). D4R are involved in relapse, thus their inhibition has been proposed as a potential strategy for addiction (Di Ciano et al., 2014).

### Schizophrenia

The “DA theory” for a dysfunction of DAergic transmission represents the first pathogenetic hypothesis of psychosis, being postulated following the fortuitous discovery of antipsychotics, acting as D2R antagonists. Actually, a hyperactivation of DAergic mesencephalic nuclei associated to a DAergic hypofunction in PFC have been demonstrated in schizophrenia (Howes and Kapur, 2009; Perez-Costas et al., 2010; Yoon et al., 2013; Weinstein et al., 2017). Besides DAergic dysfunctions, alterations in glutamatergic transmission occur, with the “DA-Glutamate hypothesis” representing the current pathogenetic theory for schizophrenia (Laruelle et al., 2003).

Supersensitivity to DA, due to modified DAergic receptors expression and/or functions, might contribute to schizophrenic symptomatology (Seeman et al., 2005).

Evidence regarding modifications in D1R expression in schizophrenia are contrasting, reporting either decreased (Okubo et al., 1997; Friedman et al., 1999) or increased levels (Abi-Dargham et al., 2002, 2012). However, hypostimulation of cortical D1R likely contributes to cognitive/negative symptoms (Abi-Dargham and Moore, 2003).

Some studies have highlighted altered D2R expression (Frankle and Laruelle, 2002; Nikolaus et al., 2009), with a specific increase in high-affinity D2R (D2RH) possibly mediating DA supersensitivity underlying psychosis (Seeman, 2011). Nevertheless, other evidence refutes modified D2R expression, supporting the idea that the alterations detected in patients represent a compensation to prolonged D2R antagonism with antipsychotics (Calabresi et al., 1992a; Weinstein et al., 2017).

Dysfunctions in the D2R-β-arrestin interaction and in β-arrestin-dependent modulation of Akt/GSK3 pathway might be involved in schizophrenia (Beaulieu et al., 2009). Actually, drugs affecting D2R/β-arrestin interaction demonstrate antipsychotic effects in animal models of schizophrenia (Park et al., 2016), revealing alternative therapeutic strategies, downstream to D2R.

In light of their restricted localization in limbic areas, D3R has been proposed as a valuable target for schizophrenia treatment (Gurevich et al., 1997). D3R modulation could improve cognitive/negative schizophrenic symptoms, without producing extrapyramidal/motor effects as D2R antagonists (Joyce and Millan, 2005). Actually, novel antipsychotics acting as D3R partial agonists/antagonists, ameliorate cognitive/negative schizophrenic symptoms (Leggio et al., 2016).

D4R also received interest as targets for schizophrenia’s treatment, since the atypical antipsychotic, clozapine, mainly acts as D4R antagonist. Moreover, increased cortical D4R levels (Seeman et al., 1993) and D4R genetic variations have been associated to schizophrenia (Hwu et al., 1998; Ptáček et al., 2011).

## Conclusion

DAergic receptors play key roles in physiological brain functioning, since they regulate locomotion, reward, cognitive functions and goal-oriented behaviors. Modifications in DAergic receptors expression and signaling occur in different neurological and neuropsychiatric disorders. While modulators of DAergic receptors already represent valuable drugs for the symptomatic treatment of PD and schizophrenia, an in-depth understanding of DAergic dysfunctions might lead to identify novel biological targets to profoundly change the fate of DA-related neurological and psychiatric conditions.

## Author Contributions

AL and NBM equally contribute in writing the article.

## Conflict of Interest Statement

The authors declare that the research was conducted in the absence of any commercial or financial relationships that could be construed as a potential conflict of interest.
